# Triple Phosphorescence and Rapid Spin Flip Mediated by Ultrafast Electron Transfer in a Spirocyclic Molecule

**DOI:** 10.1021/acs.jpclett.6c02223

**Published:** 2026-08-31

**Authors:** Jerzy Karpiuk, Elena Karpiuk

**Affiliations:** Institute of Physics, 86906 Polish Academy of Sciences, Al. Lotników 32/46, 02-668 Warsaw, Poland

## Abstract

We report triple phosphorescence emitted by sp^3^-C linked dimethylaniline-phthalide dyads (DMA-PD) and explore its mechanisms. In 77 K organic solvent glasses, ultrafast photoinduced electron transfer populates in DMA-PD a charge-separated state (CSS), with a DMA cation and PD anion. Depending on glass polarity, the anion is produced in one of two forms, with a closed or open lactone ring. The closed ring CSS deactivates to a locally excited or charge transfer triplet, while the open ring form relaxes to the zwitterion triplet state. All three triplets have different electronic configurations and are formed in different, kinetically noncorrelated intersystem crossing (ISC) mechanisms, particularly rapidly in methylcyclohexane glass (*k*_isc_ ≥ 2.3 × 10^11^ s^–1^). Our findings open the way to structural control over ultrafast triplet generation, population of multiple emissive triplet states in a single molecule, and probing heterogeneous structures and dynamics of supercooled liquids and glasses.

## Graphical abstract

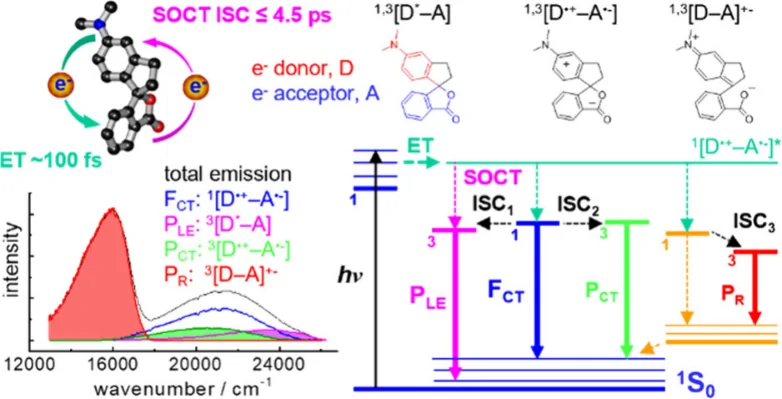

The electron spin is a crucial factor in molecular processes, and mechanisms enabling spin flip and generation of triplet states in heavy-atom-free organic molecules play a fundamental role in charge separation (CS),^1^ photovoltaics,^2^ photon upconversion,^3^ thermally activated delayed fluorescence (TADF),^4^ photocatalysis,^5^ photodynamic therapy,^6^ or magnetoreception.^7^ Nevertheless, controlled generation of triplet states, especially multiple triplets,^8^ in organic molecules is still in its infancy and requires a deeper understanding of intersystem crossing (ISC) processes, exploration of new pathways of triplet formation, and suitable molecular structures.

An interesting perspective is provided by donor–acceptor (D–A) dyads with an sp^3^ carbon atom link. They rigidly lock D and A at close proximity, and their orthogonal D–A geometry enables effective coupling of the electron orbital angular momentum with spin. Especially important for triplet generation are spirocyclic molecules, as they allow the orbital angular momentum change induced by charge recombination to be flexibly compensated by a spin flip to ensure angular momentum conservation.^9^ Suitable sp^3^-linked D–A structures enable ultrafast electron transfer (ET) and lead to highly accelerated ISC processes and dual phosphorescence.^10–14^

Here, we report spin transformations in a compact spirocyclic D–A dyad composed of dimethylaniline (DMA) and phthalide (PD) moieties, MGLA-C5 (Scheme 1),^15^ in 77 K organic solvent glasses, unveiled by single fluorescence and triple phosphorescence, wherein the latter term denotes emission composed of three phosphorescence bands from three different triplet species. Using time-resolved emission spectroscopy on μs to s time scales, we discovered an anomalous dependence of the 77 K luminescence on glass polarity and assigned observed phosphorescence bands to three different triplet states of three different species with different electronic configurations, populated in kinetically noncorrelated ISC processes based on different mechanisms. Comparison with a non-spirocyclic analogue, MGLA^16^ (Scheme 1), shows that DMA–PD dyads represent a class of D–A molecules with ultrafast charge transfer-mediated spin flip controlled by fine structural details and electric properties of the environment.

**Scheme 1:**
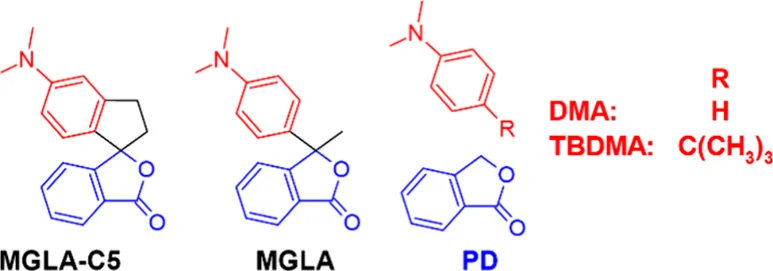
Spirocyclic (MGLA-C5) and Non-spirocyclic (MGLA) DMA–PD Dyads with D and A Subunits

Solutions of MGLA-C5 were studied at 295 and 77 K by steady-state and time-resolved luminescence in methylcyclohexane (MCH), butyronitrile (BTN), and propionitrile–butyronitrile mixture (PRN–BTN, 4:5, *v/v*) and compared with MGLA. The experimental details and procedures are described in the Supporting Information. At 295 K, the photoexcitation in the first absorption band populates in both molecules a locally excited ([^1^D*–A]) state on the DMA moiety, but no emission from it is observed. Instead, a highly solvatofluorochromic fluorescence band (Figure 1A) proves, as for malachite green lactone,^11^ an ultrafast (∼100 fs)^12^ intramolecular ET from DMA to PD to form a highly polar charge-separated state (CSS) with a structure of a covalently linked radical ion pair (RP), ^1^[D^•+^–A^•–^], and with dipole moments of 26.8 (MGLA-C5) and 24.8 D (MGLA).^15,16^

**Figure 1:**
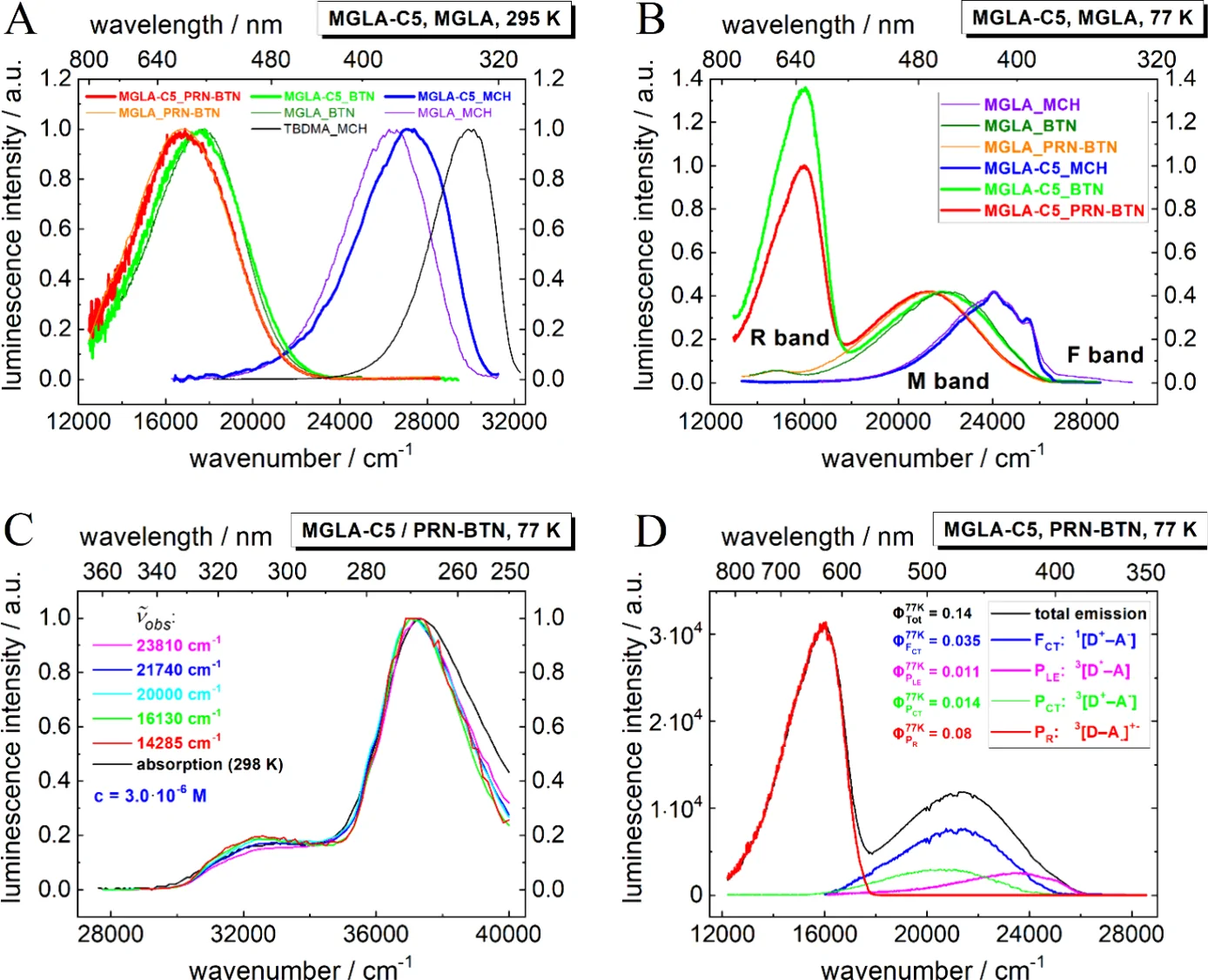
(A) Steady-state luminescence spectra of MGLA-C5 (thick line) and MGLA (thin line) in MCH, BTN, and PRN–BTN compared with TBDMA (*tert*-butyl-*N*,*N*-dimethylaniline) in MCH at 295 K and (B) at 77 K. (C) Normalized luminescence excitation spectra of MGLA-C5 in PRN–BTN at 77 K. (D) Steady-state luminescence spectrum of MGLA-C5 in PRN–BTN at 77 K decomposed quantitatively into fluorescence (F_CT_) and three phosphorescence components (P_LE_, P_CT_, and P_R_), with quantum yields of total and component emissions.

When the MGLA-C5 solution is rapidly cooled to 77 K, still no fluorescence from the photoexcited [^1^D*–A] state is emitted, indicating that the ^1^[D^•+^–A^•–^] state is populated on a time scale similar to that at 295 K. In addition, an unusual glass dependence of the luminescence spectrum is observed (Figure 1B). In MCH, MGLA-C5 displays a single luminescence band (M, ν̃_*max*_ = 24050 cm^–1^), which coincides with the M band emitted by MGLA; unlike in MGLA, however, it is not accompanied by a lower intensity F band, interpreted in MGLA as fluorescence from the ^1^[D^•+^–A^•–^] state.^10^ A considerable red shift and change in the M band shape and ν̃_*max*_ is observed in BTN (21850 cm^–1^) and PRN–BTN (21250 cm^–1^). In both polar glasses, the M band is accompanied by a high-intensity red band (R, ν̃_*max*_ = 16000 cm^–1^), reported for MGLA^10^ as a low peak at 14800 cm^–1^. While the glassochromism of the M band in MGLA-C5 mimics that observed for MGLA, the R band emitted by the spiro structure is dramatically increased and the M/R band intensity ratio is glass dependent.

The dependence of luminescence intensity at 77 K on the excitation intensity (Figure S1) proves the one-photon one-molecule origin of the observed emissions. The spectrum of MGLA-C5 in MCH does not change with the excitation energy. The M/R band intensity ratio is slightly dependent on the excitation energy in BTN and PRN–BTN. The luminescence excitation spectra show some deviation from the room-temperature absorption spectrum at 32800–37000 cm^–1^ in MCH (Figure S2) and BTN but agree well in PRN–BTN (Figure 1C).

In nonpolar MCH at 295 K, the ^1^[D^•+^–A^•–^] fluorescence of MGLA-C5 maximizes at 27400 cm^–1^ compared with 26400 cm^–1^ for MGLA (Figure 1A), wherein the higher transition energy in MGLA-C5 likely results from higher energy of the ^1^[D^•+^–A^•–^] state due to a rigidized structure. Both compounds have identical radiative rate constants, *k*_*r*_^295*K*^ = 1.8 × 10^7^ s^–1^, but show considerably different nonradiative rate constants: *k*_*nr*_^295*K*^, 19.8 × 10^8^ and 2.8 × 10^8^ s^–1^, respectively.^10,15^ The larger *k*_*nr*_^295*K*^ in MGLA-C5 indicates that the spirocyclic D–A arrangement efficiently enhances nonradiative processes in DMA–PD dyads at room temperature.

In the MCH glass at 77 K, MGLA deactivates mostly radiatively by fluorescence from the ^1^[D^•+^–A^•–^] (Φ_*F*_*CT*__^77*K*^ = 0.05, F band, Figure 1B) and phosphorescence from the DMA-localized [^3^D*–A] states (Φ_*P*_*LE*__^77*K*^ = 0.66, M band).^10^ The [^3^D*–A] state is populated by charge recombination in a spin–orbit charge transfer (SOCT) ISC mechanism^17^ from the vibronically relaxed ^1^[D^•+^–A^•–^] state with *k*_*isc*_^77*K*^ ≈ 2.3 × 10^8^ s^–1^, wherein the change in orbital angular momentum can compensate for spin flip.^10^

In contrast to MGLA, the steady-state luminescence spectrum of MGLA-C5 in MCH at 77 K does not comprise any observable fluorescence band (F) from the ^1^[D^•+^–A^•–^] state and instead coincides with the 50 ms gated emission spectrum of MGLA-C5, as well as with phosphorescence spectra of MGLA and *tert*-butyl-*N*,*N*-dimethylaniline (TBDMA) in MCH (Figure S3). This coincidence demonstrates that the luminescence of MGLA-C5 in MCH glass consists almost entirely of phosphorescence and provides a clear spectroscopic fingerprint for the DMA-localized [^3^D*–A] state as the source of the emission. In this assignment, we prefer to compare MGLA-C5 luminescence with TBDMA and not DMA, as TBDMA imitates para carbon substitution to DMA in the MGLA-C5 structure. The estimated upper limit for the fluorescence quantum yield of MGLA-C5 in MCH at 77 K, Φ_*F*_*CT*__^77*K*^ ≤ 10^–4^, was determined by integrating the dark current signal in the spectral region of the F band^10^ and comparing the integrated intensity with that of the M band. The absence of fluorescence is equivalent to rapid nonradiative deactivation of the ^1^[D^•+^–A^•–^] state. Assuming, as for MGLA, that in MGLA-C5 a vibrationally excited ^1^[D^•+^–A^•–^]* state (Figure 5) is quantitatively formed from the photoexcited [^1^D*–A] state, and *k*_*r*_^77*K*^ is the same as *k*_*r*_^295*K*^,^10^ *k*_*nr*_^77*K*^ = *k*_*r*_^77*K*^/Φ_*F*_*CT*__^77*K*^(1 – Φ_*F*_*CT*__^77*K*^) ≥ 2.3 × 10^11^s^–1^ is 3 orders of magnitude higher than in MGLA, and the nonradiative deactivation competes with vibrational relaxation and takes place from vibrationally nonrelaxed levels. As the [^3^D*–A] state is the only observed product of the ^1^[D^•+^–A^•–^] deactivation, *k*_*nr*_^77*K*^ is identified with the spin conversion rate constant *k*_*isc*_^77*K*^, and the charge recombination in ^1^[D^•+^–A^•–^] to form the [^3^D*–A] state represents an ultrafast spin–orbit charge transfer ISC. A gradual linear increase of ln­(*k*_*nr*_^295*K*^) with ^1^[D^•+^–A^•–^] energy in the low-polarity region (Figure S5) suggests that rapid acceleration of *k*_*nr*_^77*K*^ in MGLA-C5 in MCH is not likely to result from intersystem crossing to a higher locally excited triplet, but is rather related to a combination of electric characteristics of the MCH glass^18^ with the spirocyclic structure effect on the spin–orbit coupling in the SOCT-ISC process.

**Figure 2:**
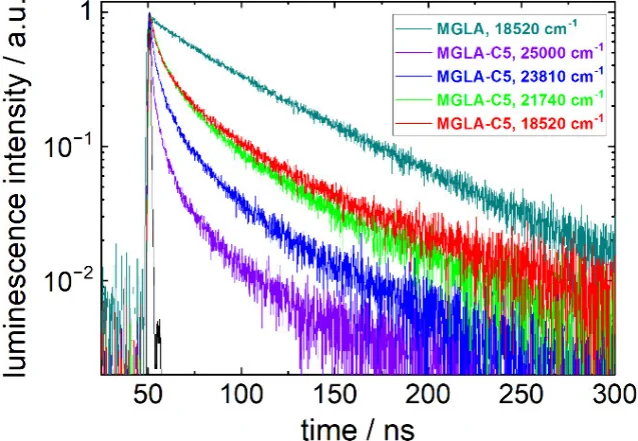
Fluorescence decays of MGLA-C5 in BTN glass at 77 K compared with MGLA (ν̃_*exc*_ = 33000 cm^–1^/303 nm).

**Figure 3:**
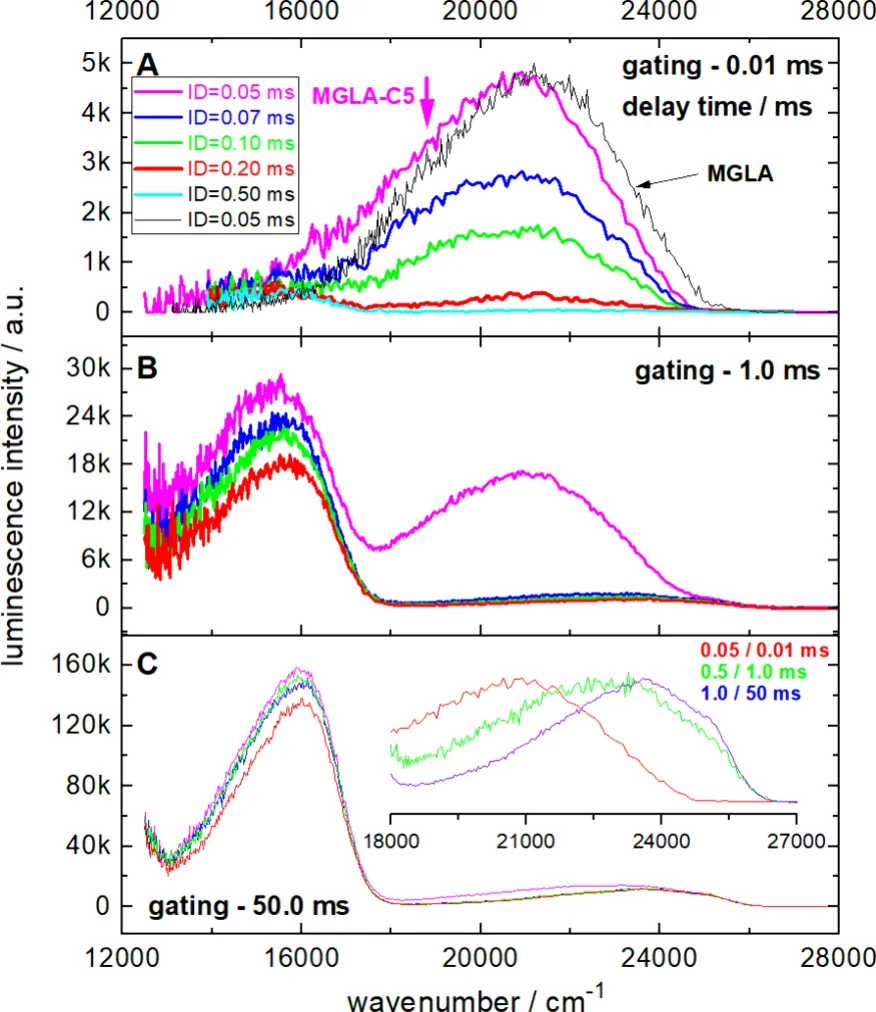
Time-resolved phosphorescence spectra of MGLA-C5 in BTN at 77 K (3 μs excitation, ν̃_*exc*_ = 32260 cm^–1^/310 nm), recorded at different initial delays (ID) with gating time *t*_*g*_: 0.01 ms (A), 1.0 ms (B), 50 ms (C); one line color marks the same initial delay in parts (A), (B), and (C). The inset in (C): normalized M band recorded at ID = 0.05, 0.5, or 1.0 ms with gating 0.01, 1.0, and 50 ms, respectively. For comparison, part (A) shows a 0.01 ms gated luminescence spectrum of MGLA in BTN at 77 K recorded after ID = 0.05 ms and assigned to the ^3^[D^•+^–A^•–^] state of MGLA.^10^

**Figure 4:**
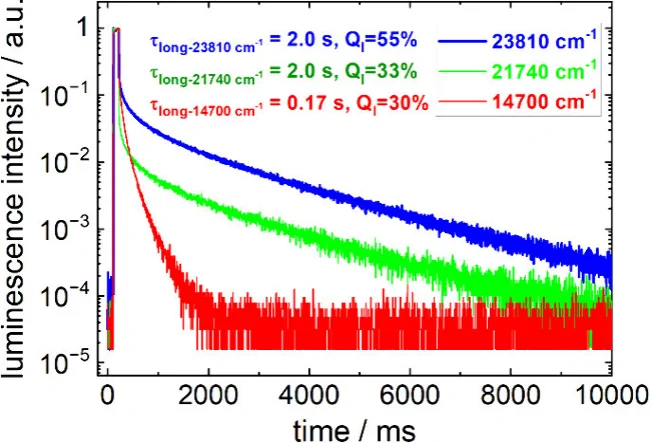
Phosphorescence decays of MGLA-C5 in BTN recorded in P_LE_, P_CT_, and P_R_ bands with the longest decay component and its contribution to a 4-exponential fit. MGLA-C5 concentration in luminescence measurements at 77 K was 1 × 10^–5^ M or less (ν̃_*exc*_ = 32680 cm^–1^/306 nm).

**Figure 5:**
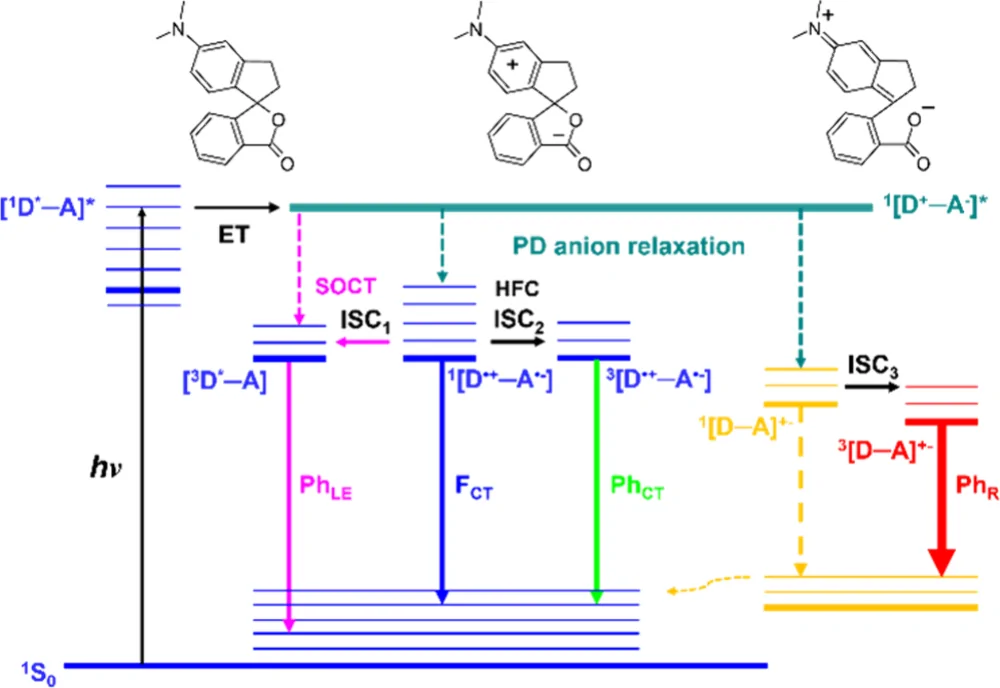
Energy diagram for MGLA-C5.

Time-resolved luminescence measurements show that the M band emitted by MGLA-C5 in polar glasses (BTN, PRN–BTN) at 77 K comprises luminescence decaying on different time scales. Unlike in MCH, a substantial portion of the M band decays on the nanosecond time scale as prompt fluorescence from the ^1^[D^•+^–A^•–^] state (Figure 1D). The fluorescence decay curves show no observable buildup, which, together with the absence of fluorescence from the photoexcited state, provides additional argument that also at 77 K the ^1^[D^•+^–A^•–^] state is populated on the ∼100 fs time scale.^11,12^ The fluorescence decay curves recorded in the M band are highly nonexponential, considerably faster than in MGLA, and emission energy-dependent, with higher-energy emission decaying significantly faster than the lower-energy one (Figure 2).

The time-resolved luminescence (TRL) spectra allow identifying the long-lived (μs to s) contribution to the M band as composed of two phosphorescence bands emitted on two different time scales (Figure 3A–C). The emission recorded with 0.01 ms gating (ν̃_*max*_ = 20850 cm^–1^ in BTN) decays entirely within 0.2 ms (Figure 3A), is spectrally similar to fluorescence, but shows no symptoms of delayed fluorescence^19^ and is similar to the MGLA spectrum recorded under identical conditions.^10^ Based on these observations and comparison, the emission is assigned to a charge transfer triplet state, ^3^[D^•+^–A^•–^], generated by hyperfine coupling (HFC) induced singlet–triplet mixing,^10^ which is indicative of a radical pair mechanism of singlet–triplet conversion.^20^

The phosphorescence persisting on the time scale of seconds (ν̃_*max*_ = 23650 cm^–1^ in BTN, long-lived component τ_long_ = 2.0 s, Figure 4) is spectrally virtually identical with phosphorescence of TBDMA in BTN at 77 K (Figure S4) and hence is assigned to the DMA-localized triplet state, [^3^D*–A]. The *k*_*nr*_^295*K*^ dependence on solvent polarity (Figure S5) suggests that in polar glasses the [^3^D*–A] state is formed from the vibrationally relaxed ^1^[D^•+^–A^•–^] state by SOCT-ISC, although a rapid spin flip from the ^1^[D^•+^–A^•–^]* state cannot be excluded as a mechanism responsible in part for [^3^D*–A] generation. The TRL spectra of MGLA-C5 reveal that [^3^D*–A] coexists with the ^3^[D^•+^–A^•–^] state since photoexcitation, similarly as in MGLA,^10^ which confirms that these triplets are formed in different, independent ISC mechanisms.

The inset in Figure 3C shows clearly the phosphorescence components of the M band and illustrates how the band shape and its dominant component evolve in time and how they depend on the gating time and initial delay used in time-resolved luminescence measurements. This dependence seemingly is similar to a time-dependent blue shift of the spectrum, but in fact is merely a consequence of considerably different radiative rate constants of the emissions from the ^3^[D^•+^–A^•–^] and [^3^D*–A] states which are observed using different initial delays and gating times.

The R band is well separated from the M band in the steady-state luminescence spectrum (Figure 1B) and comprises only the long-lived luminescence (Figure 4). Its relatively high quantum yield (Φ_*P*_*R*__^77*K*^, Table 1) and low transition energy indicate emission from an extended π-conjugated electronic system. In structurally related phthalide-based D–A dyads, rhodamine lactones (LR), such a system is formed upon adiabatic photodissociation of the C_spiro_–O bond and assigned to the zwitterionic form.^10,14,21–24^ Based on similarity with phosphorescence of triphenylmethane dyes^25–27^ and LR,^14,24,28,29^ the R band in MGLA-C5 is assigned to the triplet state of the zwitterion, ^3^[D–A]^+–^, formed upon dissociation of the C–O bond. This assignment refines our previous proposal^10^ and is related to our recent findings for LR.^14^ Though no fluorescence from the singlet state of the zwitterion, ^1^[D–A]^+–^, was detected in MGLA-C5 and MGLA, its possible involvement as an intermediate in ^3^[D–A]^+–^ generation (Figure 5) cannot be excluded, especially in view of its subpicosecond formation^30^ and solvent polarity induced ^1^[D–A]^+–^ deactivation kinetics in LR.^24^ The ^3^[D–A]^+–^ decay is not kinetically correlated with the decays of the ^3^[D^•+^–A^•–^] or [^3^D*–A] triplets (Figure 4), which confirms that all three triplets are populated on separate, glass-dependent routes, following the branching in the nonrelaxed ^1^[D^•+^–A^•–^]* state.

**Table 1:** Fluorescence and Phosphorescence Quantum Yields for MGLA-C5 and MGLA in 77 K Glasses and at 295 K

	Compound					
	MGLA-C5			MGLA^b^		
Solvent glass	MCH	BTN	P–B^a^	MCH	BTN	P–B^a^
Quantum yield						
Φ_*F*_*CT*__^295*K*^	0.009^c^	0.006^c^	0.004^c^	0.058	0.005	0.003
Φ_*Tot*_^77*K*^	0.32	0.23	0.14	0.71	0.59	0.66
Φ_*F*_*CT*__^77*K*^	≤10^–4^	0.045	0.035	0.05	0.31	0.39
Φ_*P*_*LE*__^77*K*^	0.32	0.025	0.011	0.66	0.10	0.09
Φ_*P*_*CT*__^77*K*^	≤0.001	0.016	0.014	<0.005	0.15	0.15
Φ_*P*_*R*__^77*K*^	0.0	0.144	0.08	0.0	0.03	0.03

a P–B = PRN–BTN.

b Data from ref 10.

c Data from ref 15.

Based on the results of steady-state and time-resolved measurements, the steady-state luminescence spectra were quantitatively decomposed into fluorescence and phosphorescence contributions (Figure 1D, Table 1) using the following procedure for spectral decomposition. The shapes of phosphorescence bands emitted from the locally exited triplet ([^3^D*–A]), P_LE_, and the zwitterion form (^3^[D–A]^+–^), P_R_, are obtained from the long-gated (*t*_*g*_ = 50 ms) TRL spectra recorded at longer initial delays (ID ≥ 0.5 ms), so under conditions, when only these two well-separated emissions are displayed (Figure 3C). The quantitative contribution of the P_LE_ emission to the M band in the steady-state spectrum is determined, similarly as for MGLA,^10^ using the intensity of the shoulder at 25450 cm^–1^ as a measure of the P_LE_ band intensity in the steady-state spectrum, under the assumption that mainly the donor-centered triplet state emits phosphorescence in that spectral region. To determine the P_CT_ and F_CT_ spectral contributions to the remaining portion of the M band, the long-gated (*t*_*g*_ = 50 ms) TRL spectrum recorded with an initial delay (ID) corresponding to the moment when P_CT_ has completely decayed (ID_2_ = 0.5 ms) is subtracted from the long-gated (*t*_*g*_ = 50 ms) TRL spectrum recorded with an ID as short as possible to obtain fluorescence-free luminescence (ID_1_ = 0.05 ms, cf. Figure 3C). In view of the long decay times of the [^3^D*–A] and ^3^[D–A]^+–^ states (2.0 and 0.17 s, respectively), their decays within 0.5 ms after excitation are negligible, so that the P_LE_ and P_R_ contributions to both TRL spectra are virtually identical and canceled by subtraction, and the resulting differential spectrum, P_CT_(diff), represents the emission entirely decaying between ID_1_ and ID_2_. The P_T_(diff) spectrum is found to perfectly agree in shape with the TRL spectrum recorded with ID = 0.05 ms and *t*_*g*_ = 0.01 ms (Figure 3A) and is interpreted as P_CT_ phosphorescence.

Having established the shape of the P_CT_ emission, we use the above-mentioned TRL spectra to obtain the quantitative P_CT_ contribution to the steady-state luminescence spectrum. It is done by comparing the P_CT_(diff) band with the P_R_ band in the spectrum recorded at ID_1_ = 0.05 ms and *t*_*g*_ = 50 ms, with the intensities of both bands corrected by fractions corresponding to the portions of their total intensities recorded within the gating time of 50 ms, 0.05 ms after starting the 3 μs excitation pulse. The correction is necessary, as due to different radiative rate constants of the emitting states, the intensities of individual emission bands forming the TRL spectrum are dependent on the specific ID and *t*_*g*_ and do not reflect the relative contributions of these bands in the steady-state luminescence spectrum. The intensity fraction for a given band is determined by integrating the respective part of the luminescence decay curve between ID_1_ and ID_1_ + *t*_*g*_ and dividing it by the integrated intensity of the whole decay. Subsequently, the superposition of the P_CT_ and P_R_ emission bands corrected in that way is normalized to the intensity of the R band in the steady-state luminescence spectrum and, together with the contribution from the P_LE_ emission determined as described above, subtracted from the steady-state luminescence spectrum. The resulting difference represents quantitatively the fluorescence contribution, F_CT_. The MGLA-C5 luminescence spectrum in PRN-BTN at 77 K decomposed in fluorescence (F_CT_) and three phosphorescence components (P_LE_, P_CT_, and P_R_) is shown in Figure 1D.

Table 1 compares the luminescence quantum yields of MGLA-C5 spectral components with those of MGLA in MCH, BTN, and PRN–BTN at 77 K. Similarly to that in MGLA, the total, Φ_*Tot*_^77*K*^, fluorescence, Φ_*F*_*CT*__^77*K*^, and phosphorescence quantum yields, Φ_*P*_*LE*__^77*K*^, Φ_*P*_*CT*__^77*K*^, and Φ_*P*_*R*__^77*K*^, determined with the above decomposition procedure are strongly glass-dependent, but, except for Φ_*P*_*R*__^77*K*^ in polar glasses, significantly lower than in MGLA. Fluorescence decays of MGLA-C5 are distinctly faster than in MGLA, which together with lower Φ_*F*_*CT*__^77*K*^ reflects the enhanced nonradiative deactivation of the ^1^[D^•+^–A^•–^] state in the spirocyclic structure. Considering higher Φ_*P*_*LE*__^77*K*^ and Φ_*P*_*CT*__^77*K*^ values and longer decays of P_LE_ and P_CT_ emissions in MGLA in polar glasses at 77 K, the same spirocyclic structure effect can be attributed to the corresponding triplet states in MGLA-C5.

The results reported and interpreted above clearly demonstrate the presence of single fluorescence and triple phosphorescence from a single molecular architecture in organic solvent glasses at 77 K. All these phosphorescence emissions appear after an ultrafast transformation of the locally excited singlet state [^1^D*–A] to a highly polar charge separated ^1^[D^•+^–A^•–^] state, which subsequently, entirely or in part, is converted into one of the three emissive triplet states at different stages of its vibrational relaxation. Our earlier results for MGLA indicate^10^ that the coexistence of ^3^[D^•+^–A^•–^] and [^3^D*–A] states, produced on two independent ISC pathways in the photogenerated covalently linked radical ion pair, results from operation of two mechanisms: hyperfine coupling-induced ISC and spin–orbit charge transfer ISC, respectively. In MGLA-C5, the HFC-induced ISC leading to the ^3^[D^•+^–A^•–^] triplet is even more likely, as the orthogonal, rigidly locked arrangement of DMA and PD moieties contributes to the minimization of the singlet–triplet exchange energy, i.e., the energy separation between the ^1^[D^•+^–A^•–^] and ^3^[D^•+^–A^•–^] states, to the level enabling the loss of the initially pure singlet character of ^1^[D^•+^–A^•–^] and its fast mixing with the ^3^[D^•+^–A^•–^] state. The absence of any kinetic (e.g., precursor–successor) relationship between ^3^[D^•+^–A^•–^] and [^3^D*–A] in polar glasses rules out spin-selective conversions between these triplet states and confirms their status as independent products of singlet state deactivation. The formation of [^3^D*–A] by charge recombination clearly points to SOCT-ISC as the generation mechanism of this triplet state.

Rapid nonradiative deactivation of the ^1^[D^•+^–A^•–^] state in MCH glass prevents MGLA-C5 from fluorescing, and the extremely high rate constant of the ^1^[D^•+^–A^•–^] → [^3^D*–A] conversion points to triplet formation via charge recombination from vibrationally nonrelaxed levels, indicating active involvement of vibrational excitations. The high rate of [^3^D*–A] generation in MCH is compatible with the SOCT-ISC mechanism, the rate of which depends on the relative D–A orientation and can exceed 10^10^ s^–1^ when the electron-donating and -accepting molecular orbitals are perpendicular to each other.^31,32^

It is to be noted that the ultrafast ^1^[D^•+^–A^•–^] deactivation in MCH is observed in MGLA-C5, and not in MGLA, and can be considered as a property specific to the spirocyclic D–A structure. On the other hand, relatively high fluorescence quantum yields in BTN and PRN-BTN (Table 1) and long fluorescence decays (Figure 2) confirm that both the ^1^[D^•+^–A^•–^] emission and nonradiative deactivation occur from vibrationally relaxed levels. This difference in MGLA-C5 photophysics between MCH and polar glasses indicates an active involvement of the solvent glass in vibrational relaxation of the ^1^[D^•+^–A^•–^] state and in subsequent branching into different deactivation pathways leading to three different triplet manifolds. With this in mind, the observed differences in the shape and position of the M band (including the apparent shift of the M band maximum) as well as the varying M/R band intensity ratio in BTN and PRN-BTN glasses (Figure 1B) can be explained as a manifestation of the medium influence on, primarily, the branching in the nonrelaxed ^1^[D^•+^–A^•–^]* state and subsequently the deactivation rates of all emitting species. In other words, the glassochromism of MGLA-C5 (and MGLA) is a direct consequence of changing contributions of F_CT_, P_CT_, and P_LE_ emissions to the steady-state spectrum.

The intricate relaxation of the ^1^[D^•+^–A^•–^] state, revealed primarily in nonexponential fluorescence decay curves, is likely to result from heterogeneous structures of polar glasses produced by rapid supercooling of the solution. Recently, BTN glass was reported to have a structure of a binary mixture of ordinary supercooled liquid with clusters of solid-like structures.^33^ This structural heterogeneity and differences in solvation of MGLA-C5 molecules by the mixture components would generate a broad distribution of solute environments and ^1^[D^•+^–A^•–^] states with different energies leading to different deactivation rates and pathways from the vibrationally nonrelaxed and relaxed charge-separated state. The heterogeneous glass structure can also similarly control the decay of triplet states, especially the zwitterion triplet, ^3^[D–A]^+–^, generated in polar glasses.

With the above findings, the energy level diagram proposed earlier for MGLA^10^ has been slightly modified (Figure 5). In 77 K glasses, the photoexcited ^1^[D*–A]* state deactivates by ultrafast ET to form a vibrationally nonrelaxed ^1^[D^•+^–A^•–^]* state. The high ET rate is likely related to the early dynamics of the photoexcited DMA moiety^34^ involving the interplay of its S_1_ (ππ*) state with an almost degenerated, spatially diffuse Rydberg S_2_(3s, πσ*) state.^35^ We hypothesize that in both MGLA-C5 and MGLA the driving force for ET is provided by the high dipole moment of the PD moiety (4.9 D)^36^ acting, in line with vibronic coupling, as a dipole trap, capturing electrons from the DMA orbital.^37^ In the gas phase, phthalide and phenylphthalide capture low-energy electrons via vibrational Feshbach resonance and a dipole-bound state, depositing energy selectively in C–O vibrations, thus indicating strong ET coupling with this mode.^38^ In phthalide, the resonance state transforms rapidly into two stable, vibrationally nonrelaxed anions: a short-lived closed-ring one and a long-lived open-ring one with dissociated C–O bond,^39^ and a similar mechanism with ^1^[D^•+^–A^•–^]* as a doorway to charge separation may be operative in MGLA-C5 in 77 K glasses. Our results indicate that the dipole traps and dipole-bound states can exist in the condensed phase.^40,41^

The branching into two anions leads to the excited ^1^[D^•+^–A^•–^] or ^1^[D–A]^+–^ states with closed or open lactone ring, respectively, which subsequently convert to the emitting triplets (Figure 5). The branching is controlled by the electric properties of the frozen solvent^42^ in close interplay with molecular D–A geometry, as shown by comparison of MGLA-C5 with MGLA and by preliminary results for other solvent glasses (e.g., toluene, 2-MTHF, 1-propanol). The formation of both closed and open forms is consistent with the spatially heterogeneous dynamics and structural heterogeneity of polar glasses^33,43^ originating from the coexistence of two or more liquid states or structures frozen in the glass produced from a supercooled liquid.^33^ Such competing structures in a single-component glass may differently interact with the ^1^[D^•+^–A^•–^]* and ^1^[D^•+^–A^•–^] states, thus affecting the energetic ordering and relaxation pathways of the charge-separated state manifold.

It is to be emphasized that triple phosphorescence and the underlying multiple spin conversions mediated by ultrafast electron transfer are not characteristic for a single D–A molecule but are properties of a well-defined molecular architecture with appropriately selected D and A units. DMA–PD dyads represent a class of D–A molecules with ultrafast charge transfer-mediated spin flip controlled by fine structural details and electric properties of the environment.

In conclusion, the unusual triple phosphorescence of DMA–PD dyads in 77 K solvent glasses results from a combination of ultrafast intramolecular ET, phthalide capability to form two different molecular anions, and orthogonal molecular D–A geometry. The extreme sensitivity of ET and ISC to glass polarity demonstrates that the overall triplet outcome is controlled by dyad–environment interactions, with a significant role played by electric properties and the heterogeneous structure of the surrounding medium. Our study offers thus a powerful tool for probing the dynamics of supercooled liquids and glasses. Notwithstanding the specificity of the details related to various solvent glasses, the work clearly demonstrates the unique capability of a single molecule to relax to three different triplet states of three different species with different electronic configurations. Apart from potential optoelectronic applications, molecular systems with high sensitivity to environment and populating multiple triplet manifolds may be also biologically relevant, e.g., in magnetoreception phenomena. Further research, including verification of this capability for the liquid phase and structurally related MGLA-C5 analogs, is underway, and the resulting understanding of the structure–spin conversion relationship will inspire new developments in structural engineering of triplet sensitizers, especially those based on spirocyclic architecture.

## Supplementary Material



## Notes

Experimental procedures for fluorescence and phosphorescence measurements, luminescence intensity vs excitation intensity, luminescence excitation spectra, MGLA-C5 luminescence at 77 K vs structural components, nonradiative rate constant vs solvent polarity at 295 K (PDF)
