# Supplementary material for: Triple Phosphorescence and Rapid Spin Flip Mediated by Ultrafast Electron Transfer in a Spirocyclic Molecule
Source: J Phys Chem Lett. 2026 Aug 31;17(36):10449–55. doi: 10.1021/acs.jpclett.6c02223 (PMC13573604; doi:10.1021/acs.jpclett.6c02223)
Supplement: Supplementary file 1 [file jz6c02223_si_001.pdf]

– Supporting Information –

Triple phosphorescence and rapid spin flip mediated by ultrafast electron transfer in spirocyclic molecule

Jerzy Karpiuk,[a*] Elena Karpiuk,[a]

[a] Institute of Physics, Polish Academy of Sciences, Al. Lotników 32/46, 02-668 Warsaw, Poland

Table of Contents

S1 Materials 1

S2 Experimental procedures for fluorescence and phosphorescence measurements 1

Steady state absorption and luminescence measurements at room temperature 1

Time-resolved luminescence measurements 1

Determination of luminescence quantum yields at room temperature and 77 K 2

Correction for the absorption change from 295 to 77 K 3

Correction for the refractive index change from 295 to 77 K 3

S3 Luminescence intensity of MGLA-C5 in butyronitrile glass at 77 K as a function of excitation intensity..5

S4 Luminescence excitation spectra of MGLA-C5 in MCH at 77 K compared with absorption spectrum at 295 K 6

S5 MGLA-C5 in MCH at 77 K: comparison with MGLA, TBDMA and PD phosphorescences 7

S6 MGLA-C5 in BTN at 77 K: long-gated phosphorescence at long delay time compared with TBDMA phosphorescence 8

S7 Nonradiative rate constant of MGLA-C5 as a function of solvent polarity ...9

References 10

* Corresponding author, jkarpiuk@ifpan.edu.pl

S1 Materials

5'-(dimethylamino)-3H-spiro[2-benzofuran-1,1'-indan]-3-one (MGLA-C5). MGLA-C5 was synthesized as described earlier[1], purified by column chromatography and repeatedly crystallised from a tetrahydrofuran/2-propanol mixture to give colorless transparent plates.

3,3-(dimethylaminophenyl-methyl)phthalide (MGLA). MGLA was synthesized as described earlier,[2] purified by column chromatography and repeatedly crystallized from 2-propanol.

Solvents. Methylcyclohexane (MCH, Aldrich, spectrophotometric grade) was passed through a column filled with freshly activated SiO_2 prior to use, butyronitrile (BTN, Merck, for synthesis) and propionitrile (PRN, Merck, for synthesis) were each twice distilled over $KMnO_4$ + K_2CO_3 and P_2O_5, respectively. A 4:5 *v/v* propionitrile-butyronitrile mixture (PRN:BTN) was prepared directly before use.

S2 Experimental procedures for fluorescence and phosphorescence measurements

Steady state absorption and luminescence measurements at room temperature

Absorption spectra were recorded with Shimadzu UV-2700 spectrophotometer. All steady-state luminescence spectra were recorded using a Fluorolog-3 spectrofluorometer equipped with a 450 W xenon CW lamp. The luminescence spectra were subsequently corrected for spectral response of the detection systems and additionally by subtraction of the background due to the solvent (e.g., Raman lines, reminders of the second order of the excitation line). Luminescence spectra were recorded as a function of wavelength and subsequently multiplied by factor λ^2 to convert counts per wavelength into counts per wavenumber. All luminescence measurements were carried out using solutions deoxygenated by purging with high-purity nitrogen (typically for 30 minutes before measurement).

Time-resolved luminescence measurements

Fluorescence decays on the ns time scale were recorded with Fluorolog-3 spectrofluorometer equipped with a time-correlated single-photon counting (TC SPC) Delta Hub timing electronics and a DeltaDiode excitation source (303 or 336 nm) and analyzed using the DAS6 decay analysis software package provided by Horiba Jobin Yvon (practical time resolution 0.2 ns).

Time-resolved luminescence measurements on the μs to s time scales were all carried out with a FL-1040 phosphorimeter accessory on the Fluorolog 3 spectrofluorimeter using a xenon flash lamp as the excitation source. The full-width half-maximum of the flash lamp's pulse is 3 μs, but a minimum 50 μs initial delay time was usually used to avoid any interference from the prompt fluorescence as the tail of the pulse extends to 45 μs.[3] We compared the luminescence spectra recorded with 50 and 500 ms electronic gates to verify if the former is representative for the entire long-lived luminescence and found satisfactory agreement. The signal gating procedures were performed using the spectrofluorimeter's control electronics and the FluoroEssence software.

Determination of luminescence quantum yields at room temperature and 77 K

Room temperature fluorescence quantum yields were determined using quinine sulfate in 0.1 N H_2SO_4 as a standard (Φ_f=0.51[4]) and were corrected for the refractive index of a solvent.[5] For quantum yield determinations, optical density of both the sample and the standard were identical at the wavelength of excitation, and were in the range 0.05 - 0.10. The procedure for determining the low temperature (77 K) luminescence quantum yields followed essentially that described earlier,[6] and is presented herein as adapted to MGLA-C5. The quantum yields of total luminescence, fluorescence and phosphorescence were determined for optically transparent rigid solutions (glasses) of excellent quality, with no cracks in the area illuminated by the excitation beam. For MGLA-C5 in MCH, the fluorescence spectrum of a deoxygenated sample from the stock solution in a quartz tube placed in a Dewar flask was recorded at room (295 K) temperature, and then another deoxygenated sample of the same stock solution was used for luminescence measurements at 77 K under the same conditions in the same Dewar flask filled with liquid nitrogen. Physically different samples of the same MGLA-C5 solution in MCH for measurements at 295 K and 77 K were used as the excitation of the deoxygenated sample of MGLA-C5 in MCH at room temperature during even single fluorescence scan resulted in a noticeable change in its fluorescence spectrum, likely due to photochemical generation of a fluorescent product(s). Unlike room temperature, the excitation of MGLA-C5 in MCH at 77 K did not result in any change of its luminescence spectrum and the spectra were reproducible for dozens of repeated luminescence scans, indicating that the photochemical path is inactive in the rigid MCH glass at 77 K. For MGLA-C5 solutions in BTN and PRN-BTN, the same samples were used for measurements at 295 and 77 K, as no photochemistry at RT and 77 K was observed. To determine the luminescence quantum yield at 77 K, Φ_{77K}, we followed the

procedure described by Mantulin and Huber using the following formula:[7]

$$\Phi_{77K} = \Phi_{295K}\frac{A_{295K}\, n_{77K}^2\; I_{77K}}{A_{77K}\, n_{295K}^2 I_{295K}} \tag{S1}$$

where Φ_{295K} is the fluorescence quantum yield of MGLA-C5 in a given solvent at 295 K, and *A, n*, and *I* denote absorbance, refractive index, and integral luminescence intensity of the sample, respectively, at temperature given by the subscript. According to eq. (S1), the ratio of integral intensities, I_{77K}/I_{295K}, was corrected for the change of the refractive index, *n* (n_{77K}^2/n_{295K}^2), and the change in the sample absorbance from 295 to 77 K (A_{295K}/A_{77K}) (see below).

Correction for the absorption change from 295 to 77 K

The change in absorbance of a MGLA-C5 solution on cooling from 295 to 77 K was assumed to follow the temperature-induced solvent contraction. This approach was motivated by our earlier findings in absorption measurements of MGLA in 77 K glasses,[6] indicating that the measuered absorbance change was roughly comparable to the effect of solvent contraction. For MCH, the contraction was taken to be proportional to the increase of MCH density between 293 and 77 K (from 0.77 to 1.02 g/cm^3, respectively, i.e. by factor 1.325).[8] The temperature-induced BTN contraction, expressed as the molar volume (density) change on cooling, was determined from an empirical relationship of the density as a function of temperature, extrapolated to lower temperatures:[11]

$$\varphi = \left\{ {}^{A}\!\Big/_{B^{[1+(1-{}^{T}/{}_{C})^{D}]}} \right\}^{-1} \tag{S2}$$

where: A = 0.8753, B = 0.24331, C = 582.25, D = 0.28586. The A_{295K}/A_{77K} factor for BTN obtained from eq. (S2) is 0.751.

Correction for the refractive index change from 295 to 77 K

For the refractive index correction, we used the factor n_{77K}^2/n_{295K}^2, with the *n* value at 77 K for BTN estimated according to Dannhauser and Bahe[9] from the relationship $\varepsilon_\infty = 1.1n^2$, where ε_∞ is the high-frequency dielectric costant characteristic of induced polarization. The 10% added to the square of the refractive index accounts for atomic polarization. ε_∞ for BTN was taken as 2.7,[10] and the resulting factor n_{77K}^2/n_{295K}^2 assumed in the quantum yield determination was 1.285. The n_{77K}^2/n_{295K}^2 factor for the PRN:BTN, 4:5 *v/v* mixture, could not be exactly calculated because of lack of the 77 K data for PRN and was assumed to be equal to that of BTN (1.285) because of similar values for *n* for BTN and PRN (1.3820 and 1.3658 at 293 K, respectively).[11-12] The refractive index of MCH at 77 K was calculated from the Lorentz-Lorenz equation relating the refractive index (*n*) with the density (ρ) and molecular polarizability (α)

of the solvent:

$$\frac{M}{\rho}\left(\frac{n^2-1}{n^2+2}\right) = \frac{4\pi}{3}N_A\alpha \quad \text{(S3)}$$

where *M* is the molecular weight, and N_A is the Avogadro's number. It was assumed that the molecular polarizability is independent of temperature. The n^2_{77K}/n^2_{295K} factor for MCH obtained in that way is 1.248.

S3 Luminescence intensity of MGLA-C5 in butyronitrile glass at 77 K as a function of excitation intensity

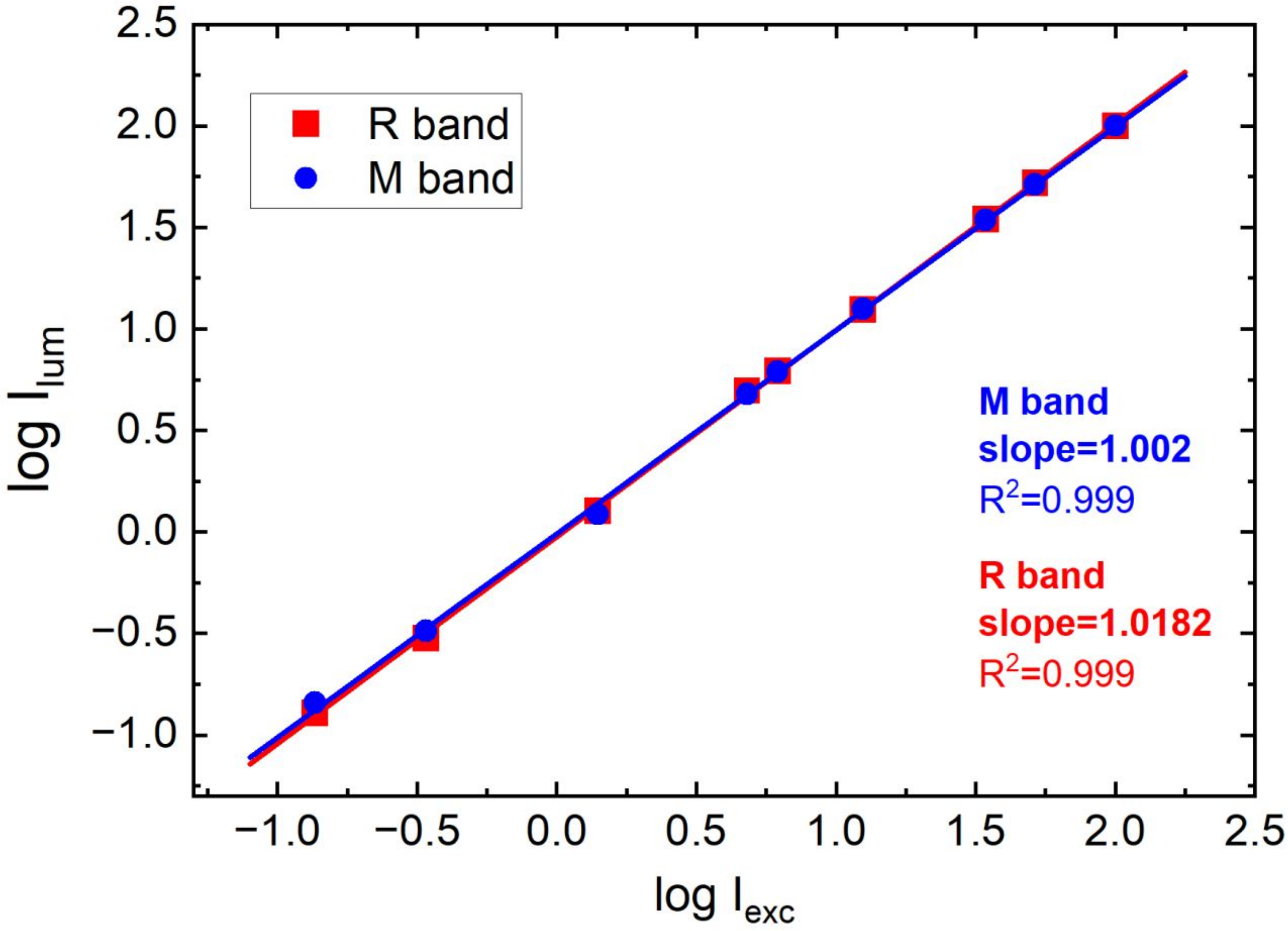

Figure S1: Luminescence intensity of MGLA-C5 in butyronitrile glass at 77 K as a function of excitation intensity: log-log plots of M band intensity (blue circles) and R band intensity (red squares). Excitation at 33000 cm^{-1} (303 nm).

S4 Luminescence excitation spectra of MGLA-C5 in MCH at 77 K compared with absorption spectrum at 295 K

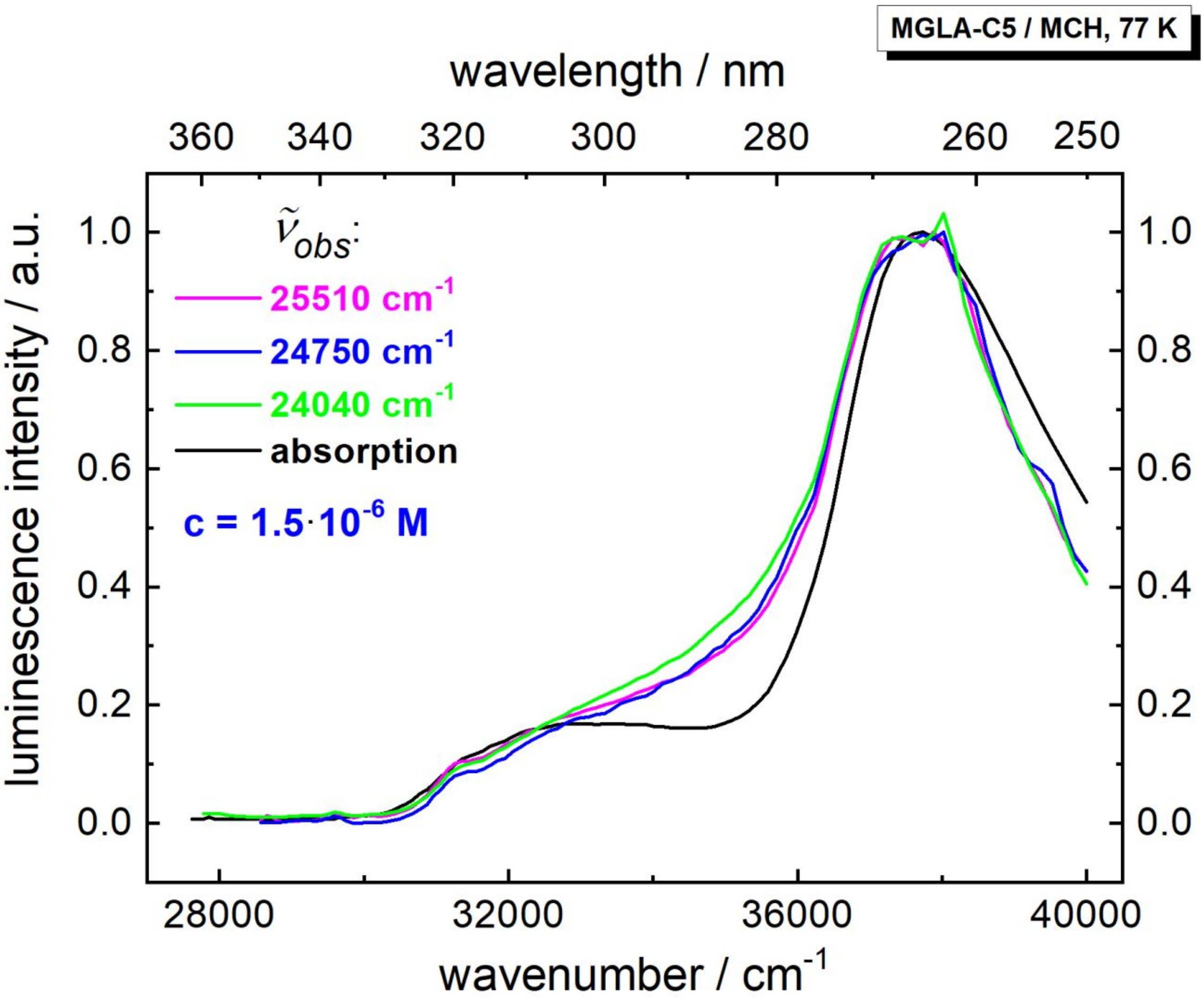

Figure S2: Luminescence excitation spectra of MGLA-C5 in methylcyclohexane (MCH) at 77 K recorded with luminescence monitored in the M luminescence band, at wavenumbers indicated in the diagram, compared with the absorption spectrum of MGLA-C5 in MCH at 295 K (black line). All spectra are normalized to the absorption maximum at 37740 cm^{-1}.

S5 MGLA-C5 in MCH at 77 K: comparison with MGLA, TBDMA and PD phosphorescences

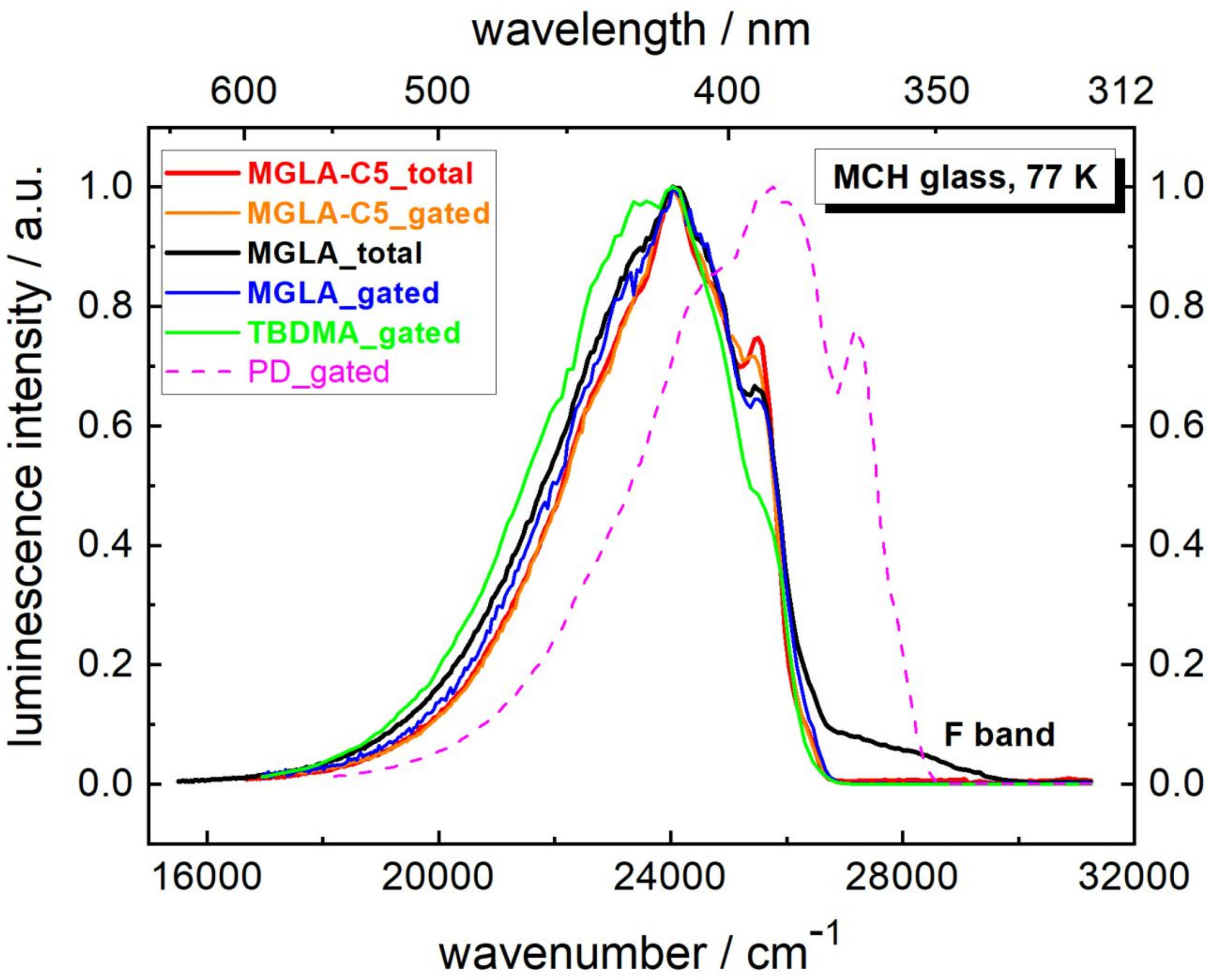

Figure S3: Normalized steady state ("total") luminescence spectra of MGLA-C5 and MGLA in methylcyclohexane (MCH) glass at 77 K compared with their phosphorescence spectra ("gated") recorded with a 50 ms gate after 50 µs delay time following a 3 µs pulse excitation at $\tilde{\nu}_{exc}$ = 32260 cm^{-1} (310 nm). The 50 ms gated phosphorescence spectrum of *tert*-butyl-N,N-dimethylaniline (TBDMA) and phthalide (PD, dashed line), recorded under the same experimental conditions, are added for comparison.

S6 MGLA-C5 in BTN at 77 K: long-gated phosphorescence at long delay time compared with TBDMA phosphorescence

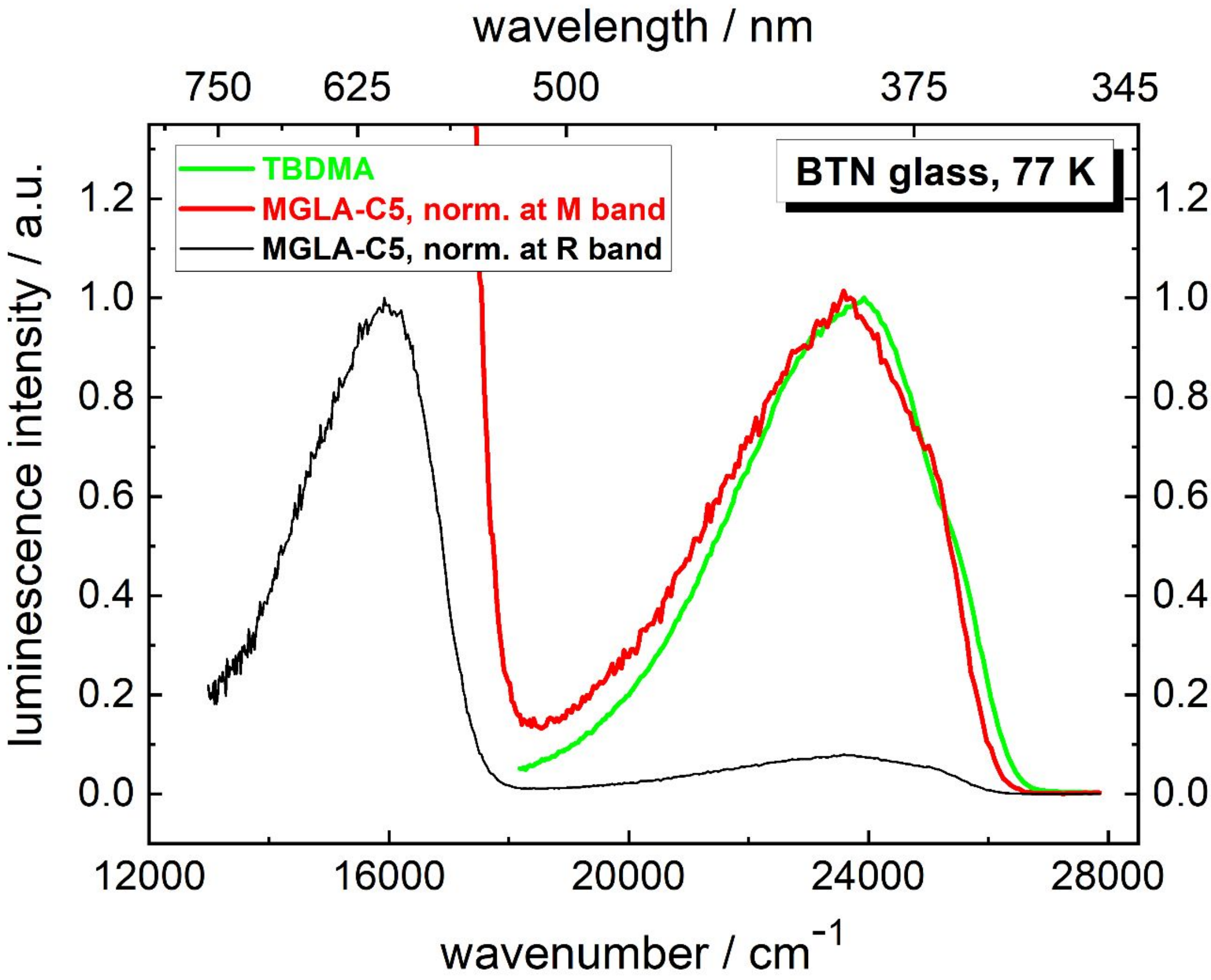

Figure S4: Time-resolved luminescence spectrum of MGLA-C5 in 77 K BTN glass (red) recorded with a 50 ms gate after 1 ms initial delay, compared with TBDMA phosphorescence in BTN glass at 77 K recorded with a 50 ms gate after 50 μs delay time, both normalized to each other at the maximum of TBDMA phosphorescence band (23920 cm^{-1}). The spectra were excited with a 3 μs pulse excitation at $\tilde{\nu}_{exc}$ = 32260 cm^{-1} (310 nm, MGLA-C5) or 35715 cm^{-1} (280 nm, TBDMA), respectively. The same MGLA-C5 spectrum normalized at the maximum of the R band (black, thin line) is added to show the proportions of the M and R bands recorded under the measurement conditions.

S7 Nonradiative rate constant of MGLA-C5 as a function of solvent polarity

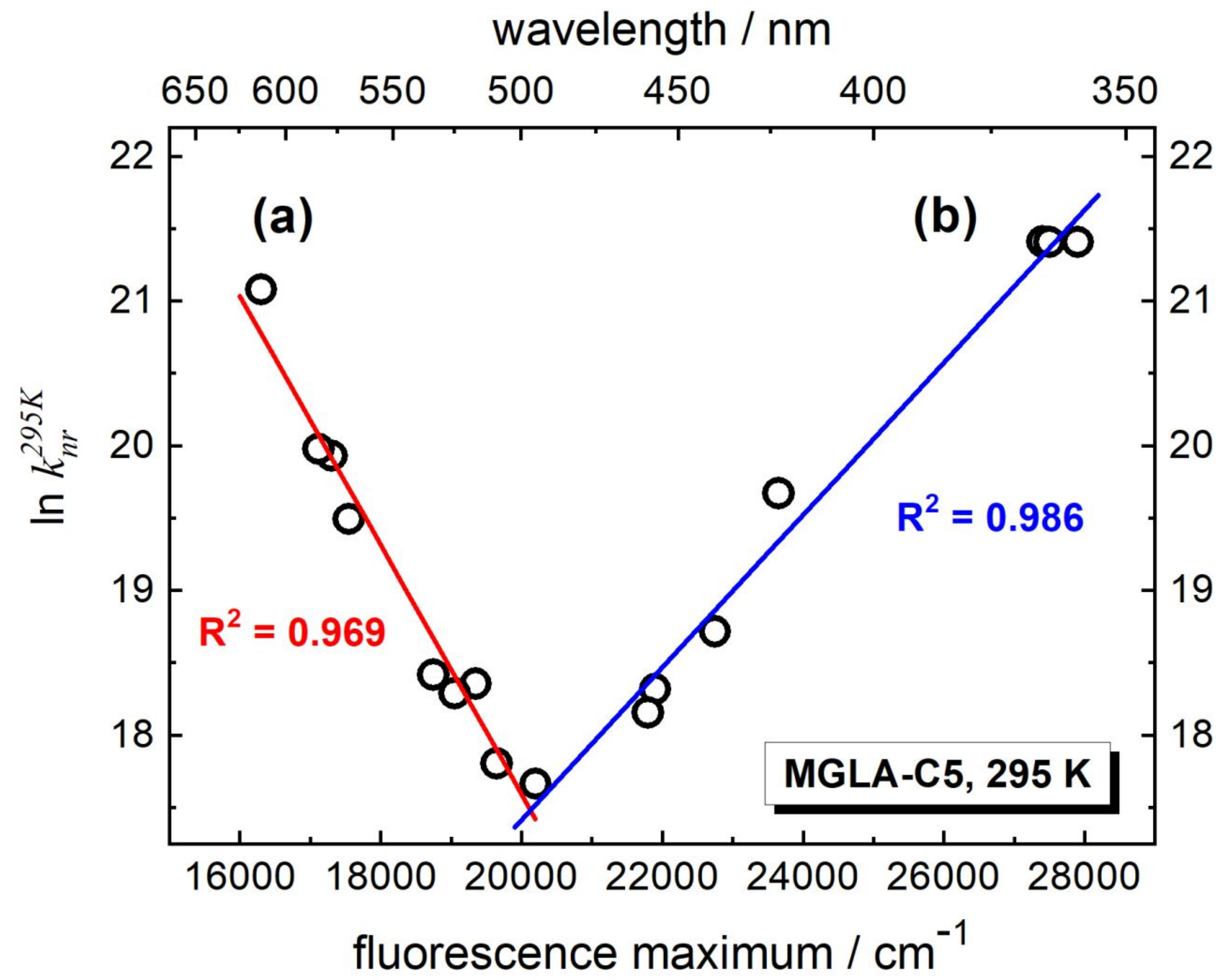

Figure S5: Logarithmic plot of nonradiative rate constant, k_{nr}^{295K}, of MGLA-C5 as a function of solvent polarity measured by the energy of $^1[D^{•+}–A^{•−}] \rightarrow S_0$ transition in MGLA-C5 (represented by the MGLA-C5 fluorescence maximum, $\tilde{\nu}_{max}$) in a given solvent at room temperature. The straight lines represent linear fits for (a) solvents with $\tilde{\nu}_{max} \leq 20000$ cm^{-1} (ethyl acetate and more polar solvents) and (b) for solvents with $\tilde{\nu}_{max} \geq 20000$ cm^{-1} (butyl acetate and less polar solvents). Coefficients of determination: R^2 = 0.98 and 0.986 for (a) and (b) straight lines, respectively. k_{nr}^{295K} data for the plot were calculated using the formula k_{nr}^{295K} = $(1\text{-}\Phi_{fl})\tau_{fl}^{-1}$) and numeric data from ref. [1].

References

[1] Karpiuk, J.; Gawryś, P.; Karpiuk, E; Suwińska, K. Electron transfer across a spiro link: extreme solvatofluorochromism of a compact spirobridged *N,N*-dimethylaniline-phthalide dyad. *Chem. Commun.* **2019**, *55*, 8414-8417.

[2] Karpiuk, J.; Karolak, E.; Nowacki, J. Photoinduced Intramolecular Charge Separation and Recombination in a Donor-Acceptor Dyad Linked via Tetrahedral Carbon Atom. Photophysics of a Malachite Green Lactone Analogue. *Pol. J. Chem.* **2008**, *82*, 865-882.

[3] *Fluorolog-3 Spectrofluorometer. FL-1040 Phosphorimeter with Dual-Lamp Housing. Operation Manual. Part number J81075 rev. C.*, Horiba Scientific, 2012.

[4] Velapoldi, R. A. Considerations on Organic Compounds in Solution and Inorganic Ions in Glasses as Fluorescence Standard Reference Materials. *Journal of Research of the National Bureau of Standards - A. Physics and Chemistry.* **1972**, 76A, 641-654.

[5] Eaton, D. F. Reference materials for fluorescence measurement. *Pure & Appl. Chem.*, **1988**, *60*, 1107-1114.

[6] Karpiuk, J; Majka, A.; Karolak, E.; Nowacki, J. Intramolecular Electron Transfer in Frozen Solvents: Charge Transfer and Local Triplet States Population Dynamics Revealed by Dual Phosphorescence. *J. Phys. Chem. Lett.* **2017**, 8, 4659-4667. Supporting Information.

[7] Mantulin, W. W.; Huber, J. R. Measurement of the Temperature Dependence of the Fluorescence Quantum Yield of Organic Molecules in Solution. *Photochem. Photobiol.* **1973**, *17*, 139-143.

[8] Kevan, L. Trapped electrons in organic glasses. *Adv. Radiat. Chem.* **1974**, *4*, 181-306.

[9] Dannhauser, W.; Bahe, L. W. Dielectric Constant of Hydrogen Bonded Liquids. III. Superheated Alcohols. *J. Chem. Phys.* **1964**, *40*, 3058-3066.

[10] Ito, N.; Duvvuri, K.; Matyushov, D. V.; Richert, R. Solvent Response and Dielectric Relaxation in Supercooled Butyronitrile. *J. Chem. Phys.* **2006**, *125*, 024504.

[11] Daubert, T. E.; Danver, R. P. *Physical and thermodynamic properties of pure chemicals*, Hemisphere Publishing Corp., New York, 1989.

[12] Reichardt, C. *Solvents and solvent effects in organic chemistry*. 3rd Edition, Wiley-VCH, Weinheim, 2003.
